# Peltomexicanin, a Peltogynoid Quinone Methide from *Peltogyne Mexicana* Martínez Purple Heartwood

**DOI:** 10.3390/molecules21020186

**Published:** 2016-02-04

**Authors:** Paulina Gutiérrez-Macías, Javier Peralta-Cruz, Amparo Borja-de-la-Rosa, Blanca E. Barragán-Huerta

**Affiliations:** 1 Department of Environmental Systems Engineering, Escuela Nacional de Ciencias Biológicas, Instituto Politécnico Nacional, Av. Wilfrido Massieu S/N, Unidad Profesional Adolfo López Mateos, Gustavo A. Madero, México City CP 07738, Mexico; pau_21_gm@hotmail.com; 2 Department of Organic Chemistry, Escuela Nacional de Ciencias Biológicas, Instituto Politécnico Nacional, Prolongación de Carpio y Plan de Ayala, S/N. Miguel Hidalgo, México City CP 11340, Mexico; javierperaltacruz@yahoo.com; 3 Division of Science Forestry, Universidad Autónoma Chapingo, Carr. Mexico-Texcoco Km 38.5, Texcoco, México State CP 56230, Mexico; aborja@correo.chapingo.mx

**Keywords:** *Peltogyne mexicana* Martínez, peltogynoids, quinone methide, purple heartwood, peltomexicanin

## Abstract

Peltomexicanin (7,10-dihydroxy-6,12-dioxa-5*H*-tetraphen-3-one) is a new peltogynoid quinone methide isolated from Palo Morado (*Peltogyne mexicana* Martínez) heartwood by column chromatography. Its chemical structure was elucidated by IR, NMR (^1^H, ^13^C), 2D NMR experiments (COSY, NOESY, HMQC, and HSQC), ESI-MS, and UV-Vis spectroscopic analysis. According to HPLC quantification, this compound is the main pigment and accounts for 1.21% of Palo Morado heartwood material. The antioxidant activity of peltomexicanin and dried methanolic extract (DEx) of purple heartwood was evaluated using the radical of 2,2’-azinobis-(3-ethylbenzothiazoline-6-sulphonic acid) (ABTS) assay, and the corresponding values expressed as Trolox equivalents (µmol TE/mg sample) were 4.25 and 4.57, respectively.

## 1. Introduction

*Peltogyne mexicana* Martínez*,* commonly known as Palo Morado, is one of the twenty-three species belonging to *Caesalpiniaceae* family, and it is an endemic tree from Guerrero State, Mexico. The principal characteristic of its heartwood is a particular purple color; native people exploit this attribute and the wood’s hardness to elaborate wooden crafts, furniture and souvenirs [1]. Previous studies in other species have shown that the compounds responsible for the color are related to peltogynoids. These compounds are derivatives of flavonoids, with which they share a similar chemical structure; they can found in fruits, barks, roots, and aerial parts from species of the genus *Peltogyne*, *Acacia*, *Caesalpinia* and *Cassine*, among others [2,3,4,5,6,7,8]. The first peltogynol isolated was (+)-peltogynol from *P. porphyrocardia* Griseb. ex Benth by Robinson and Robinson [9] and its chemical structure was elucidated by Hassall and Weatherston [10]. Peltogynin and mopanin the respective oxidation products of (+)-peltogynol and its isomer, mopanol, were reported by Brandt and Roux [4] and Drewes and Roux [11]. The compounds described in these studies have a chromophoric group in their structure that confers color. In this study, we present the isolation, structural analysis, and quantification of peltomexicanin, a peltogynoid quinone methide from the heartwood of *P. mexicana*.

Quinone methides are analogous compounds to quinones, wherein a carbonyl oxygen has been replaced by a methylene group. There are three isomers of quinone methides: *ortho-*, *para-* and *meta-,* but the *ortho-* and *para-* types are the most common. Also, quinone methides are polar and more reactive than quinone and simple enones (like α,β-unsaturated ketones). The importance of their study lies in which they are intermediates in chemical and biochemical synthesis reactions [12]. Besides, it has been reported that the *ortho*-quinone methide moieties confer important biological activities. For example, they have antioxidant properties and they are responsible for the cytotoxic and/or cytoprotective effects of many drugs, natural products, and endogenous compounds [13]. Therefore, the interest on the development of methods for quinone methides synthesis has been increased in the recent years [14], although simple quinone methides, such as, those without substituents at the exocyclic methylene group, are unstable compounds. Because of that, many efforts have been focused on their stabilization, mainly form quinone methide coordination complexes with transition metals [15,16,17]. To the best of our knowledge, however, none of the peltogynoid quinone methides have yet been characterized.

## 2. Results and Discussion

### 2.1. Structural Elucidation of the Isolated Compound

A purple amorphous powder was obtained, with UV-Vis (MeOH) λ_max_ nm (log ε) 286 nm (3.64), 398 nm (3.42), 530 nm (3.44) and 566 nm (3.37); λ_max_ (+NaOMe) 288 nm (3.58), 344 nm (3.38), 432 nm (3.46), 616 nm (3.68); λ_max_ (+HCl) 282 (3.43), 396 nm (3.05), 536 nm (3.56). Bands typical of phenolic compounds were observed in the IR spectrum of the pure pigment; these were similar to those observed in compounds related to peltogynoids [18]. We observed a stretch band at 3352 cm^−1^ characteristic of the O-H bond, two bands of stretching 2970 and 2923 cm^−1^ for the C-H bond, a signal of a carbonyl group at 1611 cm^−1^, a band at 1283 cm^−1^ characteristic of the -C-O-C= group. Aromaticity (Ar-H) of the compound was represented by an intense band at 883 cm^−1^. Pigment analysis by ESI-MS in positive mode generated a [M + H]^+^ ion at *m*/*z* 283.0589, (*m*/*z* expected 283.0601) in agreement with the molecular formula C_16_H_11_O_5_. 

Proton NMR exhibited a set of six protons in the aromatic region corresponding to aromatic protons of two independent systems A and B. A COSY experiment revealed a three coupled proton system attributed to H-8 (doublet, 7.94 ppm), H-9 (double doublet 6.25 ppm, *J* = 1, 10 Hz) and H-11 (doublet 6.33 ppm, *J* = 1 Hz) in ring A. The three protons for ring B were observed as broad singlets at 7.27 ppm (H-4), 7.16 ppm (H-2) and 6.66 ppm (H-1), respectively. Methylene protons for C-5 were observed as a broad singlet at 5.06 ppm. Correspondent protonated carbons were attributed based on results from an HSQC experiment (Table 1); carbon shifted at 158.43 ppm (C-10) was attributed to the *ipso*-hydroxy group as well as C-11a shifted at 152.58 ppm.

Fusion between rings ABCD was evidenced by long range ^1^H-^13^C HMBC correlation, in ring A the proton shifted at 6.33 ppm (H-11) has a three bond correlation with (C-9), proton H-9 (δ 6.25 ppm) in the same ring correlated with C-11 (δ 107.52 ppm). H-8 was correlated with the carbon located at 152 ppm (C-11a) which is the fused enol ether group of the system. The second hydroxyl group was found in ring C and shifted the corresponding *ipso* carbon C-7 to 146.06 ppm due its enol character.

**Figure 1:**
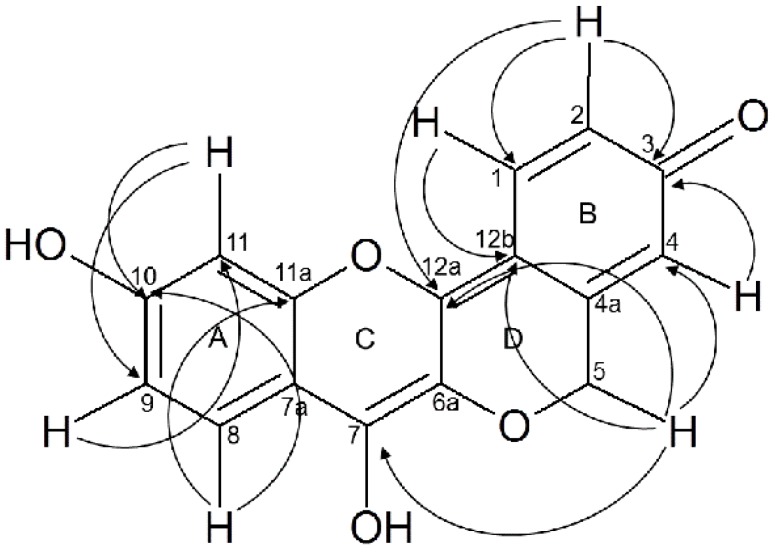
Peltomexicanin, multiple long range correlations observed by HMBC.

Methylene carbon C-5 (δ 66.64) was evidenced by ^13^C attached proton test (APT) analysis, methylene protons correlated with alpha carbon C-4 (δ 112.58) in ring B. In ring D we observed long range correlations ^1^H-^13^C between the methylene protons (δ 5.06 ppm) with the carbon C-7 (W) and C-12b belonging to the quinone methide structure conformed by ring B and ring D (Figure 1). Two bond ^1^H-^13^C correlation was observed from protons H-2 and H-4 shifted to 7–16 and 7.27 ppm respectively with the carbonyl group of quinone moiety located at 179.36 ppm in ^13^C-NMR.

**Table 1:** ^1^H- (500 MHz) and ^13^C-NMR (125 MHz) (296 K in methanol-*d*_4_) spectroscopic assignments for peltomexicanin.

Carbon Number	^13^C δ	^1^H *δ*, Multiplicity, *J* (*Hz)*	HMBC ^1^H-^13^C Correlations
1	110.94	6.66, *br s*	C-12b
2	108.76	7.16, *br s*	C-1, C-3, C-12a
3	179.36		
4	112.58	7.27, *s*	C-3
4a	159.54		
5	66.64	5.06 *s*	C-4, C-7 (W) C-12a C-12b
6a	113.16		
7	147.0		
7a	133.47		
8	131.74	7.94, *d* (10)	C-11a, C-10
9	107.52	6.26, *dd* (1, 10)	C-11
10	158.13		
11	102.50	6.33, *d* (1)	C-9, C-10
11a	152.58		
12a	146.06		
12b	121.81		

Thus, the structure was established as 7,10-dihydroxy-6,12-dioxa-5*H*-tetraphen-3-one, due the observed IR, MS and ^1^H- and ^13^C-NMR data. We named the pigment as “peltomexicanin”. The proposed structure suggests that peltomexicanin represents a rare peltogynoid quinone methide compound. 

Results obtained for pigment analysis by UV-Vis, IR, ESI-MS, and 1D/2D nuclear magnetic resonance techniques confirm that we have isolated a flavonoid not previously reported, which in its enolized form, it has a similar structure to that of peltogynin and mopanin. The molecular weights reported for those compounds are 298.05 g/mol, whereas the molecular weight determined for peltomexicanin was 282.05 g/mol, indicating that the pigment from *P. mexicana* has one less hydroxy group than peltogynin or mopanin.

### 2.2. Quantification by HPLC-DAD

The HPLC chromatogram showed a well-defined peak at 530 nm for the purified pigment with a retention time of 11.08 min. The peltomexicanin concentration calculated in the semipolar fraction was 11.85%. This represents 9.12% of the dried extract and 1.21% of the heartwood of *P. mexicana*.

### 2.3. Antioxidant Activity

The antioxidant activity of peltomexicanin expressed as Trolox equivalent antioxidant capacity (TEAC) was calculated as 1.20 ± 0.04 mM, which is lower compared to that reported for other flavonoids such as quercetin (TEAC = 3.68 ± 0.12) and myricetin (3.07 ± 0.23 mM) [19]. This may be since peltomexicanin differs from those flavonoids in that it contains a fourth ring that blocks the reactive groups, which are usually free in flavonoids. Thus, this structural configuration could result in a reduced ability of the molecule to neutralize radicals.

The specific radical scavenging estimated for peltomexicanin was 4.28 ± 0.10 µmol TE/mg; this value was slightly lower than 4.57 ± 0.10 µmol TE/mg obtained with the dried extract (DEx). This may be attributable to the presence of other compounds in the extract that could increase the antiradical activity alone or synergistically.

In conclusion, we have isolated a new purple pigment from *P. mexicana* Martinez and identified it as a quinone methide peltogynoid. This compound, named peltomexicanin, showed an important antioxidant activity. This feature lays the groundwork for additional studies of its biological activity.

## 3. Experimental

### 3.1. General Procedures

UV-Vis spectra were obtained using a DR 5000 spectrophotometer (Hach, Loveland, CO, USA) in a range from 230 to 700 nm. The IR spectrum was recorded on a Jasco FT-IR instrument equipped with ATR (Jasco, Easton, MD, USA). The exact molecular mass was obtained by (ESI) analysis on a Bruker micrOTOF-Q II instrument (Bruker Daltonics, Bremen, Germany). Samples were dissolved in methanol and were injected directly to the spectrometer. The capillary potential was −4.5 kV, the dry gas temperature 200 °C and the drying gas flow 4 L/min. Total ion chromatograms from *m*/*z* 50 to 3000 were obtained. MS data were processed using Data analysis 1.0 (Bruker Daltonics). NMR experiments (^1^H at 500 MHz) and ^13^C at 125 MHz) were performed with Varian 500 equipment (Varian, Palo Alto, CA, USA) using methanol-*d_4_* as a solvent and TMS as internal reference.

### 3.2. Plant Material

*P. mexicana* Martinez (Palo Morado) was collected in Guerrero State (17°09′32′′ N latitude and 99°30′51′′ W longitude). Plant was identified by Post-graduate College of Chapingo, Mexico and a voucher specimen (Registration number 51,392) was deposited at the Herbarium CHAP.

### 3.3. Extraction and Isolation

The heartwood of *P. mexicana* was crushed into a fine powder and then sieved through US standard testing sieve #45 to #100. Particles retained in mesh #100 (355 µm < particle size > 150 µm), were extracted in the dark with MeOH (1% HCl) in a solvent: solid ratio of 95:5 (*v*/*w*) at 20 ± 2 °C for 24 h in a 250 mL flask with magnetic stirring. The solution was then filtered and concentrated on a rotatory evaporator (R-200, Büchi, Flawil, Switzerland) at 40 °C. This yielded an amorphous purple powder, which was named “dried extract” (DEx). Prior to purification, DEx was added to a partition liquid-liquid in a system of water-ethyl acetate (EtOAc) in order to remove compounds that were either highly polar or water- and EtOAc-insoluble. One gram of DEx was dissolved in 100 mL of EtOAc and 30 mL of distilled water to neutral pH was added. This solution was mixed vigorously and then organic phase was recovered; the operation was repeated by adding an additional two volumes of water. The organic fraction (corresponding to the semipolar fraction of DEx) was concentrated with a rotatory evaporator at 40 °C. This fraction represented 76.93% ± 1.73% of DEx.

Subsequently, 600 mg of the semipolar fraction were dissolved in 2.0 mL of MeOH and loaded onto a column packed with Sephadex LH-20 (1.5 × 35 cm, LH20 Sigma, St. Louis, MO, USA). As eluent, a gradient of MeOH:H_2_O was used in proportions of 0:10, 1:9, 2:8, 3:7, 4:6, 5:5, 6:4, 7:3, 8:2, 9:1 and 10:0. The pigment fraction of interest was concentrated and dried with a rotary evaporator at 40 °C.

### 3.4. Quantification by HPLC-DAD

Quantification of peltomexicanin pigment in the heartwood was performed by HPLC analysis, using the pure pigment as standard of reference. One milligram of peltomexicanin was diluted in 1 mL of MeOH at 1, 0.8, 0.6, 0.4, and 0.2 mg/mL, and filtered with 0.2-µm pore nylon acrodisc. Sample (20 µL) was injected onto the HPLC-DAD instrument (Infinity 1260 Series, Agilent Technologies, Palo Alto, CA, USA) equipped with a quaternary pump and analyzed by a ZORBAX Eclipse Plus C18 column (Agilent Technologies) 4.6 i.d. × 150 mm, 5 µm; with a gradient elution of distilled water (phase A) and acetonitrile (phase B) at a flow of 1 mL/min, starting at 100% of phase A for 5 min, reaching 50% of phase B in 12 min, and 100% of phase B in 4 min, maintaining for 3 min gradient, returning to phase A 100% in 3 min and then held for a further 3 min. The oven temperature was set at 25 °C. Chromatographic analysis was monitored at 280 nm and 530 nm in DAD channels. A calibration curve was constructed using the area data obtained for each pigment dilution. In parallel, a sample of the semipolar fraction of DEx at 5 mg/mL was analyzed by HPLC in the same conditions as above, and the corresponding area for peltomexicanin was recorded. Using the external standard method, the peltomexicanin concentration in the extract and in the heartwood was calculated.

### 3.5. Antioxidant Activity

The antioxidant activity of the DEx and the pure peltomexicanin were determined by measuring their capacity to scavenge the pre-formed radical of 2,2’-azinobis-(3-ethylbenzothiazoline-6-sulphonic acid) (ABTS) according to the method described by Rufino *et al.* [20]. ABTS^+^ radical was produced by reacting 50 mL of 7 mM ABTS (A1888 Sigma) stock solution with 880 µL of 140 mM potassium persulfate (379824 Sigma) and allowing the mixture to stand in a dark room at room temperature for 16 h before use. The ABTS^+^ solution was diluted with methanol to obtain an absorbance of 0.70 ± 0.04 at 734 nm. Then, 1.5 mL of ABTS^+^ radical and 30 µL of sample or standard were mixed for 6 min and final absorbance was measured at 734 nm on a Hach DR5000UV-Vis spectrophotometer. Calibration was performed with a Trolox (238813 Sigma) stock solution as described above and the results were presented as TEAC.

## Supplementary Materials

The following are available online at http://www.mdpi.com/1420-3049/21/2/186/s1.
