# Supplementary material for: Peltomexicanin, a Peltogynoid Quinone Methide from Peltogyne Mexicana Martínez Purple Heartwood
Source: Molecules. 2016 Feb 4;21(2):186. doi: 10.3390/molecules21020186 (PMC6272850; doi:10.3390/molecules21020186)
Supplement: Supplementary file 1 [file molecules-21-00186-s001.pdf]

Supplementary Materials: Peltomexicanin, a Peltogynoid Quinone Methide from *Peltogyne Mexicana* Martínez Purple Heartwood

Paulina Gutiérrez-Macías, Javier Peralta-Cruz, Amparo Borja-de-la-Rosa and *Blanca E. Barragán-Huerta

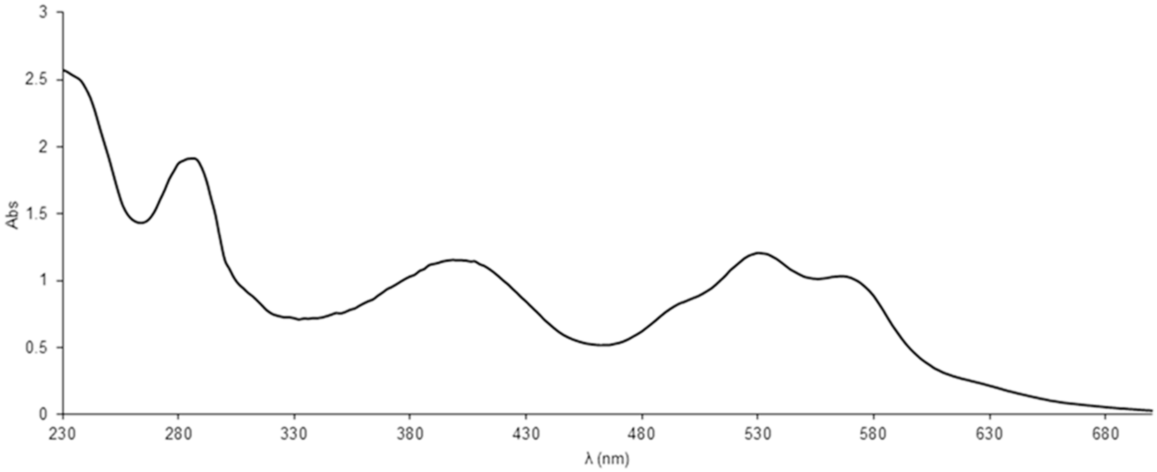

Figure S1. UV-Vis absorption spectrum of peltomexicanin (0.125 mg/mL) in MeOH.

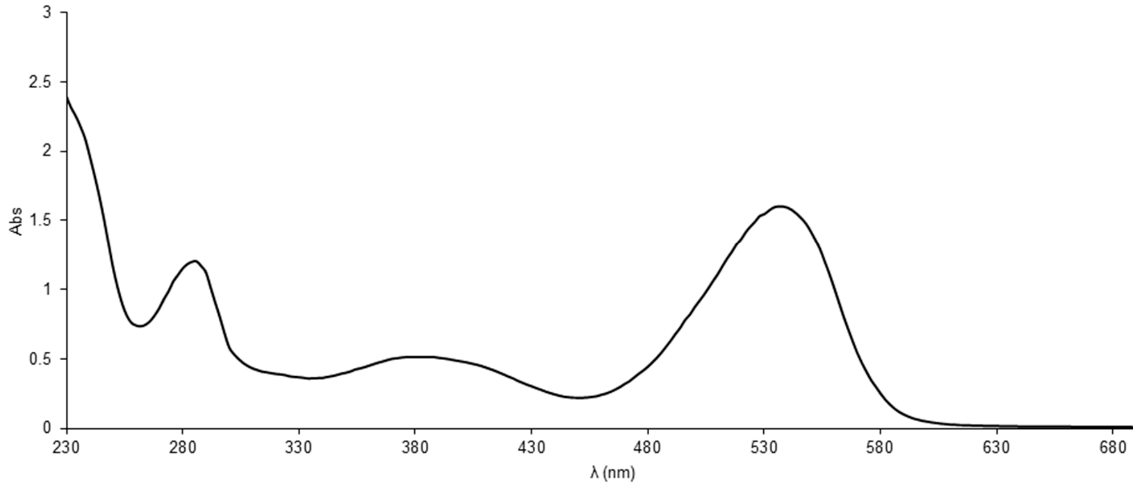

Figure S2. UV-Vis absorption spectrum of peltomexicanin (0.125 mg/mL) in MeOH + HCl (0.1 M).

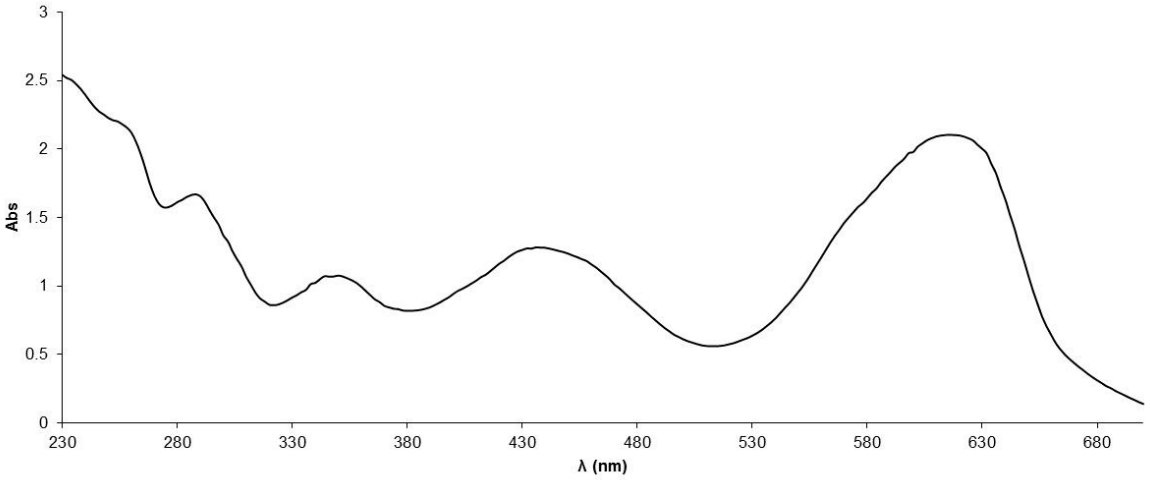

Figure S3. UV-Vis absorption spectrum of peltomexicanin (0.125 mg/mL) in MeOH + NaOH (0.1 M).

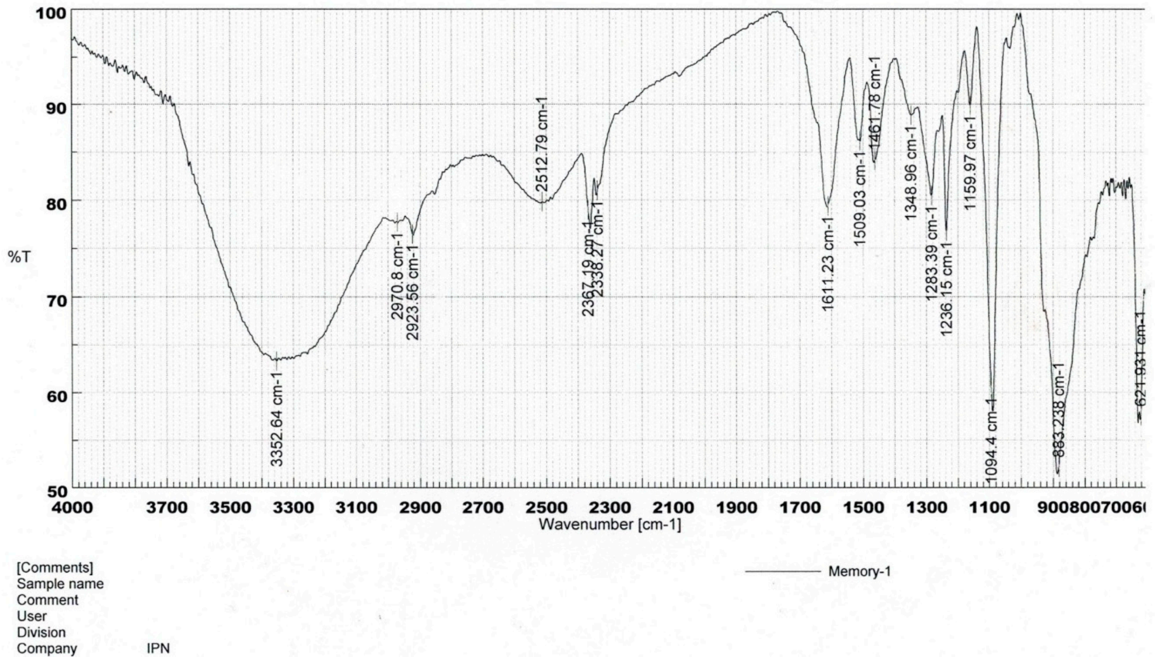

Figure S4. IR sprectrum of peltomexicanin.

Display Report

Analysis Info

Analysis Name	D:\Data\RogelioJimenez\101414_Roge008_Pos.d	Acquisition Date	10/14/2014 3:47:20 PM
Method	tune_low.m	Operator	Daniel
Sample Name	101414_Roge008_Pos	Instrument / Ser#	micrOTOF-Q II 10392
Comment			

Acquisition Parameter

Source Type	ESI	Ion Polarity	Positive	Set Nebulizer	0.4 Bar
Focus	Active			Set Dry Heater	180 °C
Scan Begin	50 m/z	Set Capillary	4500 V	Set Dry Gas	4.0 l/min
Scan End	3000 m/z	Set End Plate Offset	-500 V	Set Divert Valve	Source

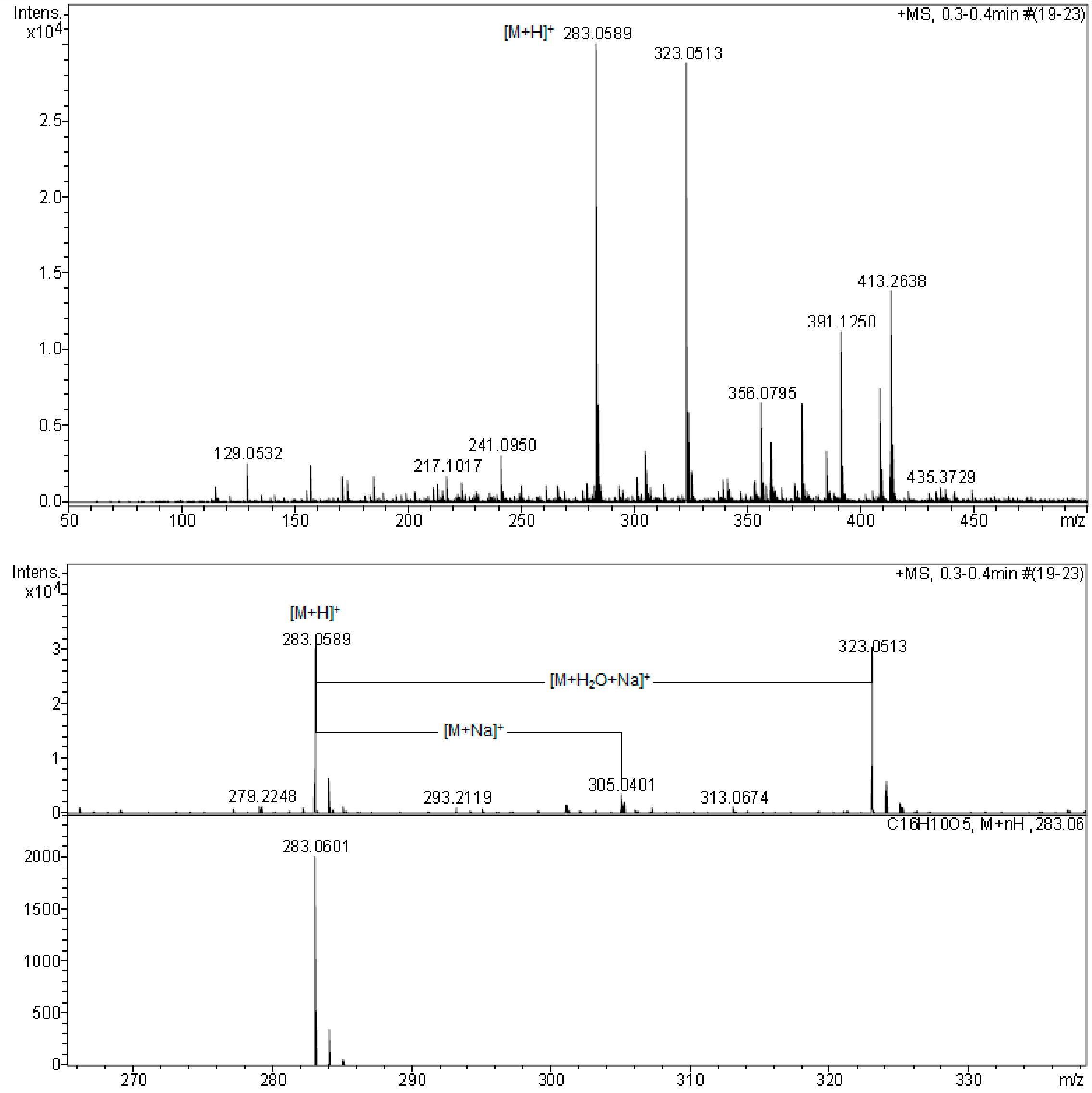

Figure S5. ESI-MS spectrum (positive mode) of peltomexicanin.

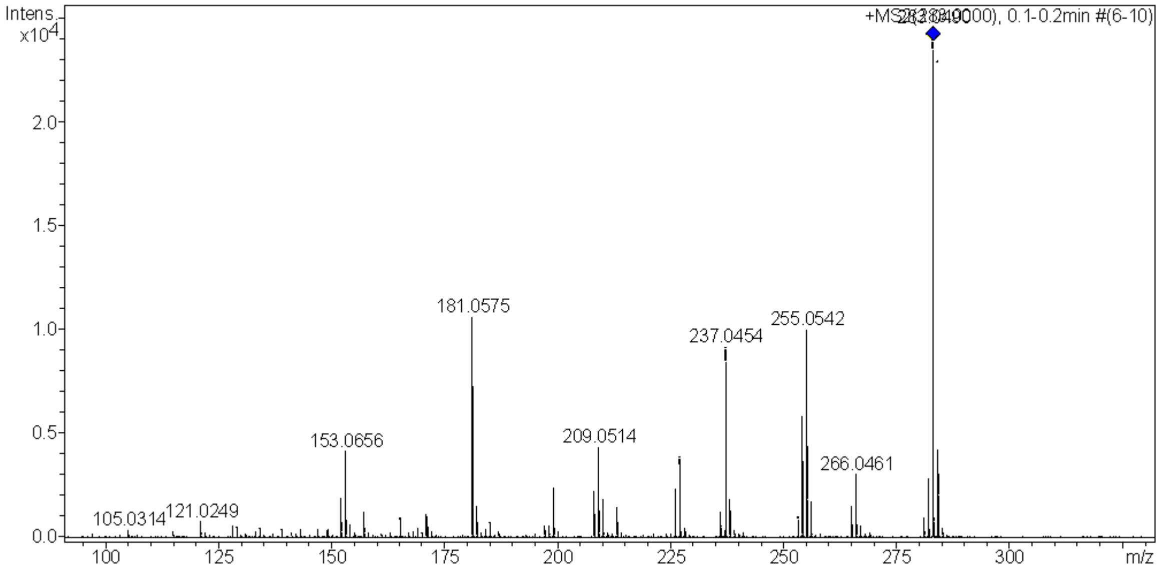

Figure S6. Spectrum MS/MS (positive mode) of [M + H]$^+$ ion at *m/z* 283.0589.

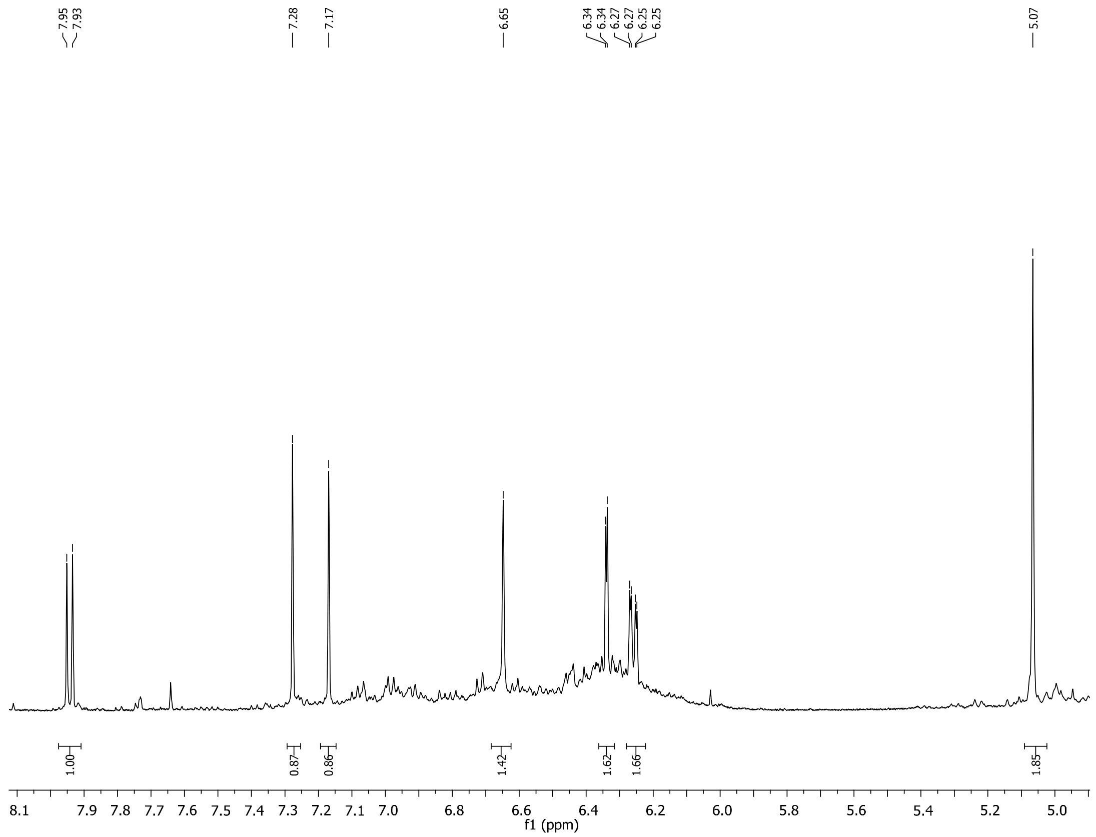

Figure S7. Spectrum ^{1}H-RMN (500 MHz, MeOH-d_4).

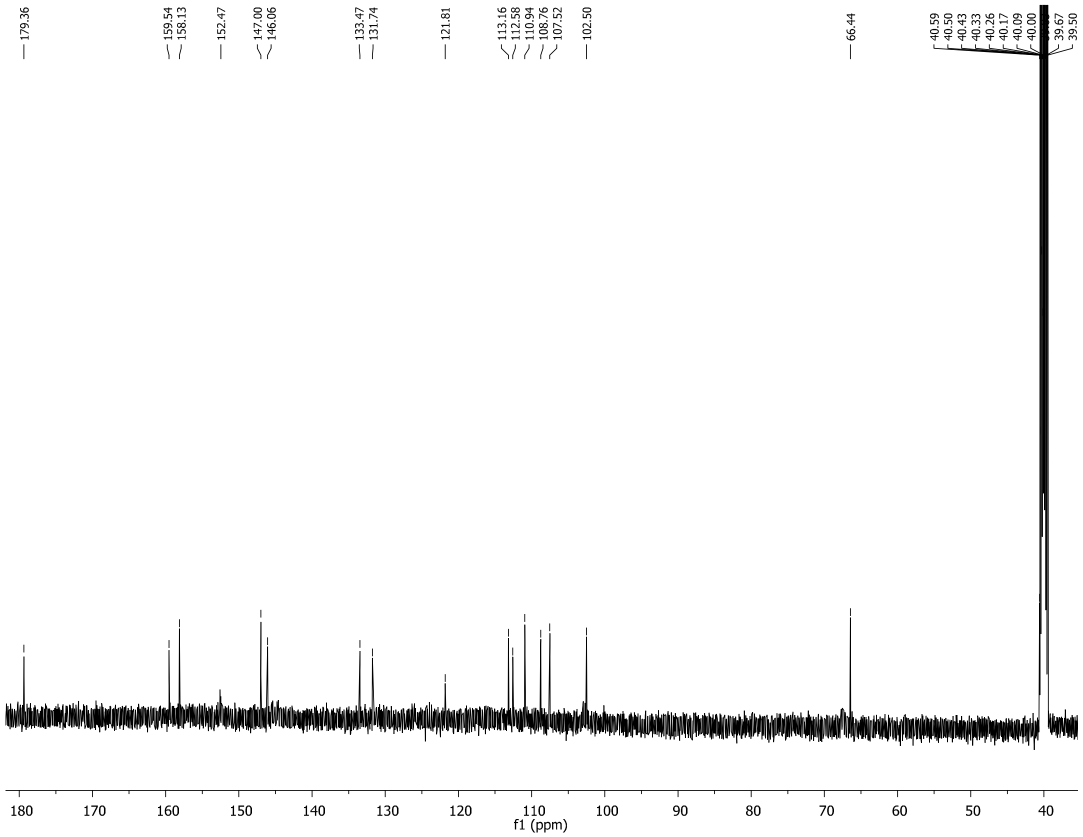

Figure S8. Spectrum ^{13}C-RMN (125 MHz,MeOH-*d*$_4$).

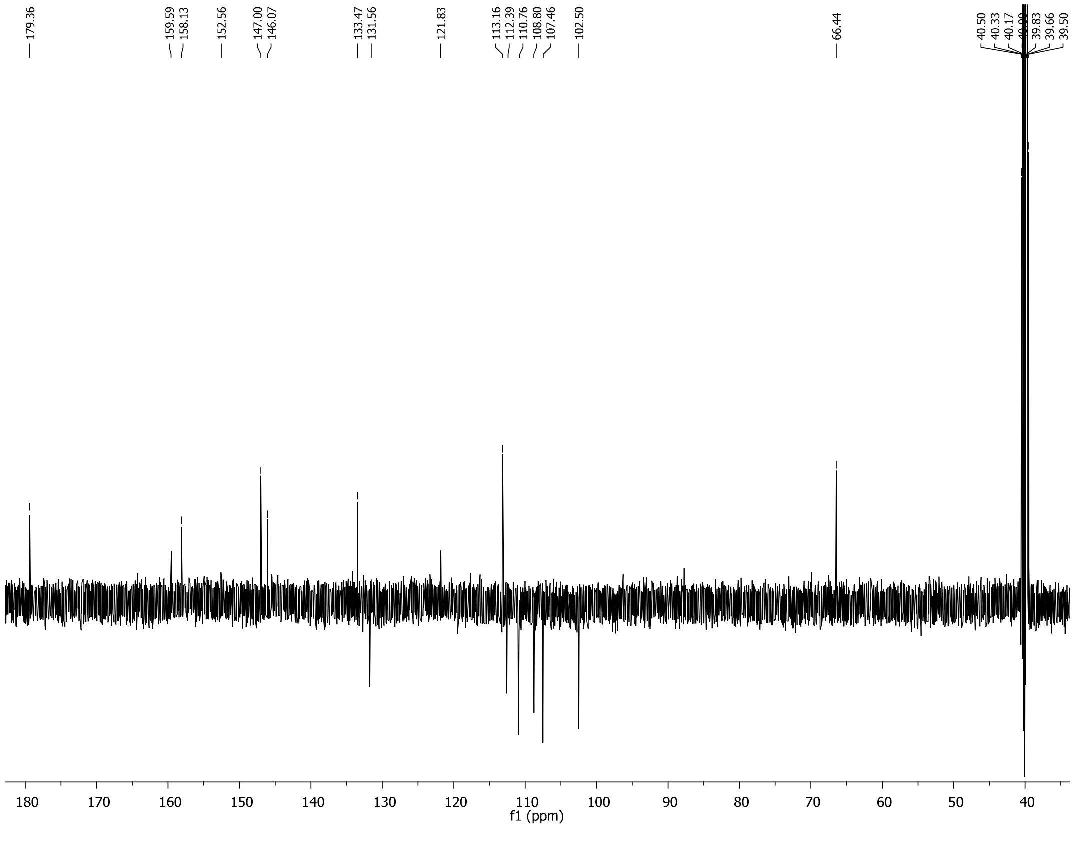

Figure S9. Spectrum ^{13}C-RMN APT (500 MHz,MeOH-*d*$_4$).

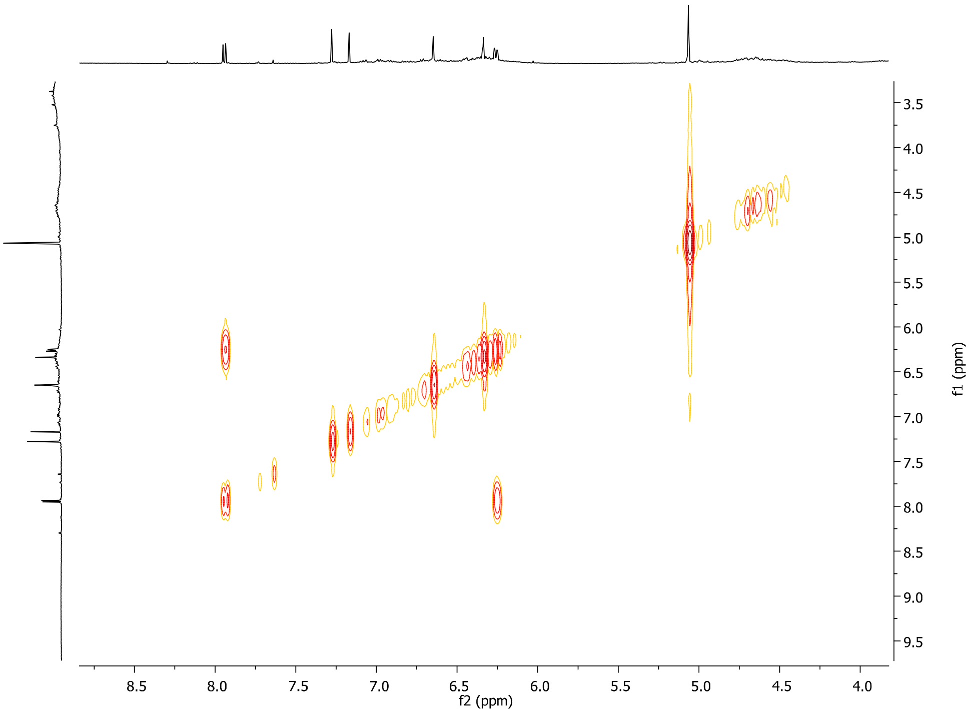

Figure S10. Spectrum COSY.

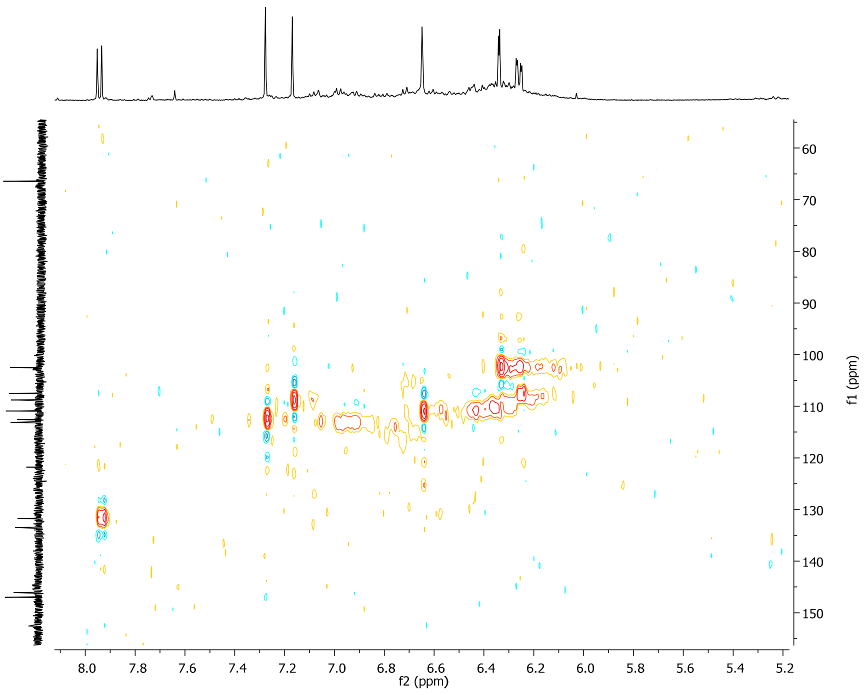

Figure S11. Spectrum HSQC.

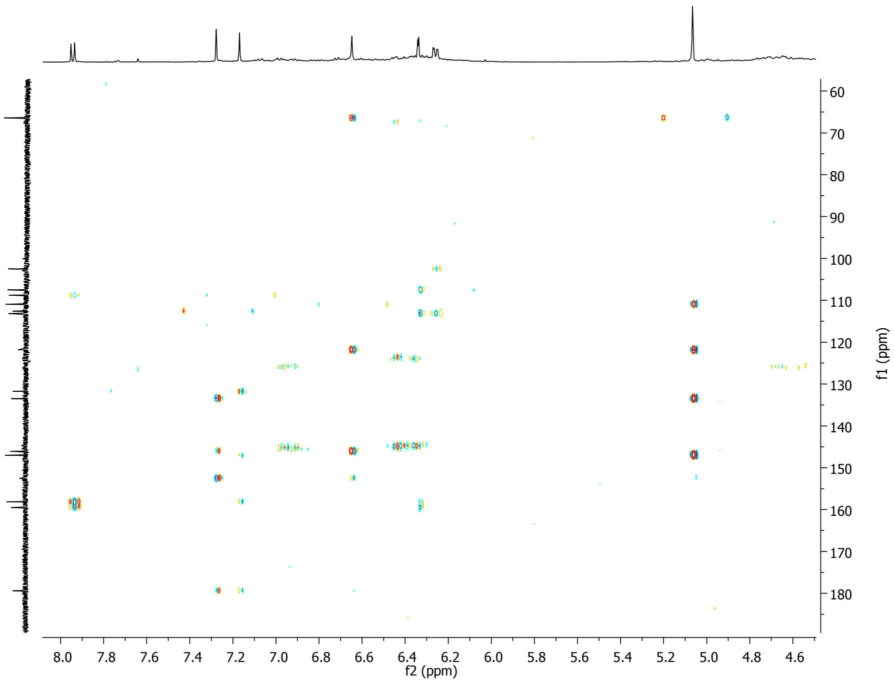

Figure S12. Spectrum HMBC.

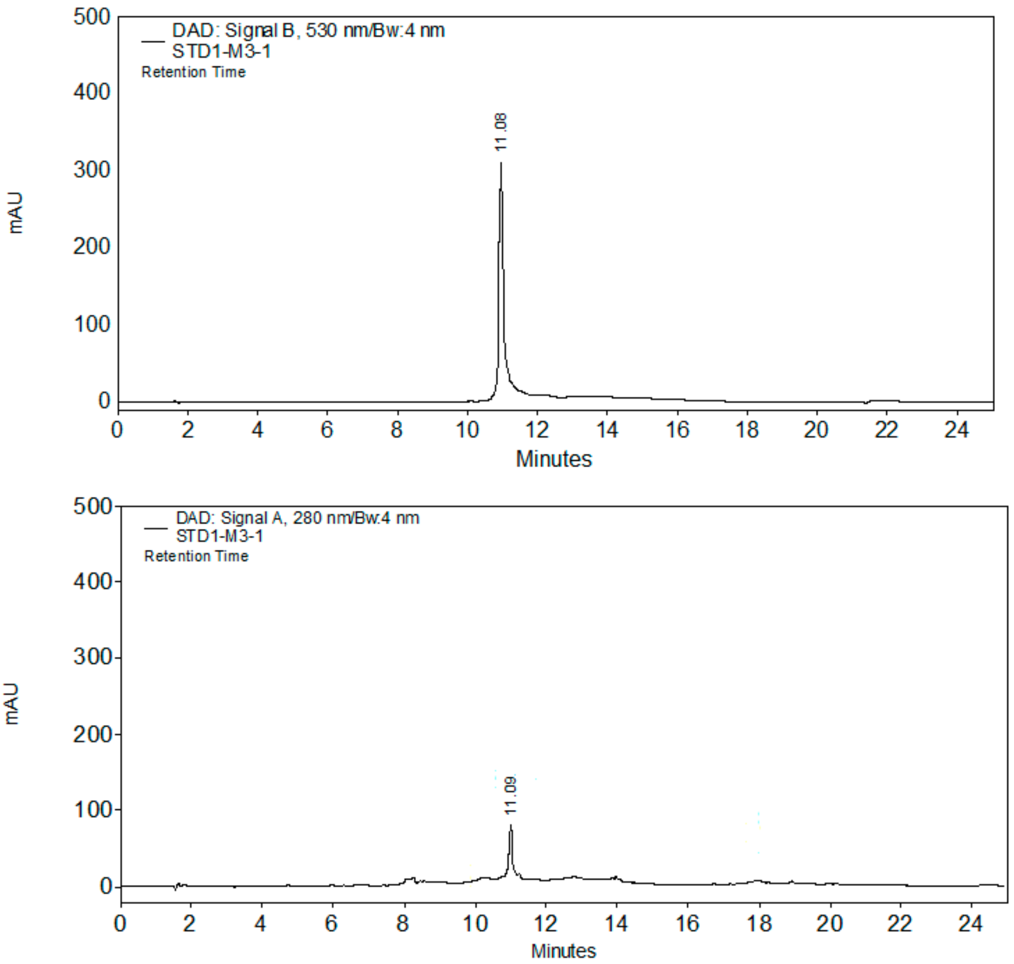

Figure S13. HPLC chromatograms of peltomexicanin (Up 530 nm, below 280 nm). Conditions: Gradient H_2O; acetonitrile; column C18 ZORBAX Eclipse Plus (150 × 4.6 mm, 5 µm), 20 µL, 1 mg·mL^{-1}, 1 mL·min^{-1}.

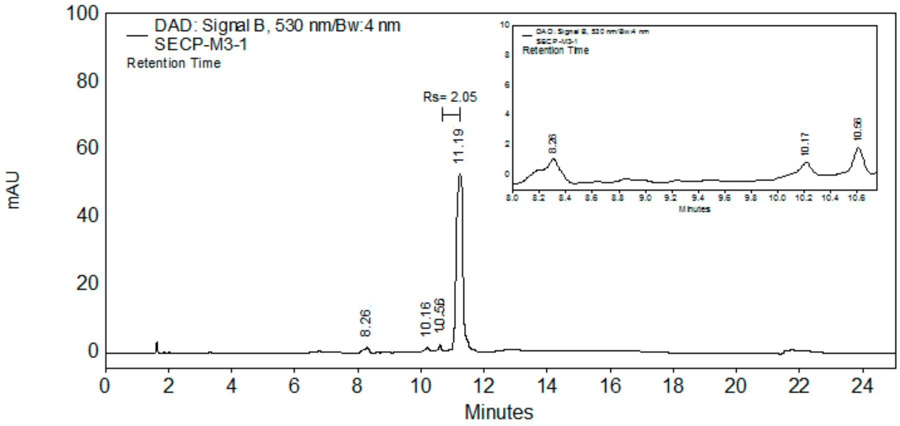

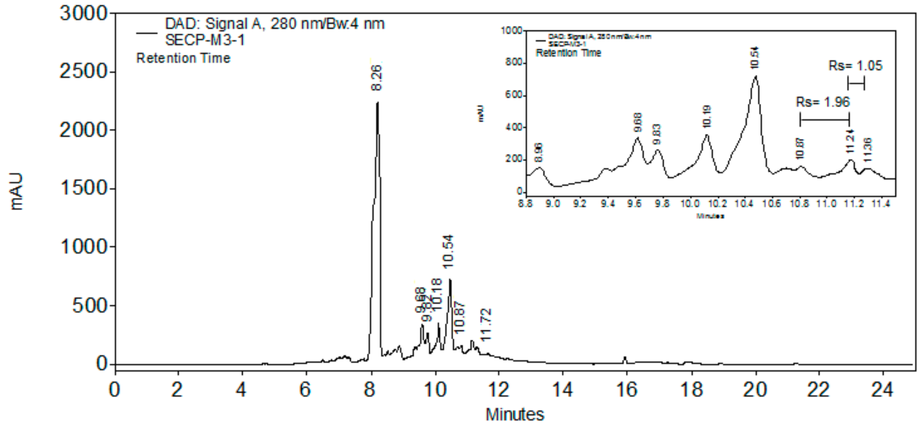

Figure S14. HPLC chromatograms of semipolar fraction of dried extract (Up 530 nm, below 280 nm). Conditions: Gradient H_2O; acetonitrile; column C18 ZORBAX Eclipse Plus (150 × 4.6 mm, 5 µm), 20 µL, 5 mg·mL^{-1}, 1 mL·min^{-1}.
